# Intra-Continental Transport of Western Wildfire Smoke Heightens Health Risks Across North America

**DOI:** 10.3390/ijerph22020226

**Published:** 2025-02-05

**Authors:** Erica D. Bruce, Akinleye Folorunsho, Nilkamal Jaisawal, Emily Gaw, Yang Li

**Affiliations:** Department of Environmental Science, Baylor University, One Bear Place #97266, Waco, TX 76798-7266, USA; akinleye_folorunsho1@baylor.edu (A.F.); nilkamal_jaisawal1@baylor.edu (N.J.); emilyagaw@gmail.com (E.G.)

## Abstract

Wildfires in North America, particularly in western states, have caused widespread environmental, economic, social, and health impacts. Smoke from these fires travels long distances, spreading pollutants and worsening the air quality across continents. Vulnerable groups, such as children, the elderly, and those with preexisting conditions, face heightened health risks, as do firefighters working in extreme conditions. Wildfire firefighters are of particular concern as they are fighting fires in extreme conditions with minimal protective equipment. This study examined wildfire smoke during July–August 2021, when intense fires in Canada and the western U.S. led to cross-continental smoke transport and caused significant impacts on the air quality across North America. Using the GEOS-Chem model, we simulated the transport and distribution of PM_2.5_ (particulate matter with a diameter of 2.5 μm or smaller), identifying significant carcinogenic risks for adults, children, and firefighters using dosimetry risk methodologies established by the U.S. EPA. Significant carcinogenic risks for adult, child, and firefighter populations due to exposure to PM_2.5_ were identified over the two-month period of evaluation. The findings emphasize the need for future studies to assess the toxic chemical mixtures in wildfire smoke and consider the risks to underrepresented communities.

## 1. Introduction

Wildfires have been a significant issue in North America over the past several years, impacting various regions, particularly the western states. Intercontinental wildfires have far-reaching impacts that extend beyond the immediate areas affected by the flames. These impacts can be environmental, economic, social, and health-related, and they can affect regions far from the origin of the fires due to the movement of smoke and pollutants across continents. The environmental impacts include air quality degradation, climate change, a loss of biodiversity, and soil and water quality issues, thus affecting aspects of social, economic, and human health [1,2,3,4,5,6,7].

Wildfire smoke can travel thousands of miles, affecting the air quality in regions far from the fire source. This smoke contains particulate matter (PM), carbon monoxide, and other toxic pollutants that can harm human health and ecosystems. Wildfires also release significant amounts of carbon dioxide (CO_2_) and other greenhouse gasses, contributing to global warming. The loss of forests also reduces the planet’s capacity to sequester carbon. Fires can destroy habitats and threaten the survival of species. The recovery of ecosystems can take decades, and some species may face extinction [4,8,9,10]. The removal of vegetation can lead to soil erosion and the loss of nutrients [3,11]. Ash and debris can contaminate water sources, affecting aquatic life and human water supplies.

In terms of economic and social impacts, wildfires can cause extensive damage to homes, businesses, and infrastructure, leading to costly repairs and rebuilding efforts. Farmland and crops can be destroyed, causing economic losses for farmers and disruptions in food supply chains. Popular tourist destinations affected by wildfires can see a decline in visitors, impacting local economies dependent on tourism. An increased frequency and severity of wildfires can lead to higher insurance premiums and greater financial risk for insurance companies [12,13,14,15,16,17,18,19].

The social and health impacts of wildfires are far-reaching and pose significant long-term health effects. Exposure to wildfire smoke can cause respiratory and cardiovascular problems, particularly in vulnerable populations such as children, the elderly, and those with preexisting health conditions. Wildfire firefighters are of particular concern as they are fighting fires in extreme conditions with minimal protective equipment [2,16,20,21,22,23,24]. Additionally, mental and psychological health impacts have been observed, often attributed to the disruption of daily life, the trauma associated with the loss of life and property, and the long-term health effects that some individuals experience long after the flames are extinguished [2,16,20,21,22,25,26,27,28]. Evacuations and the loss of homes can lead to significant psychological stress and trauma for affected individuals and communities. The destruction of infrastructure and homes can disrupt communities and lead to long-term displacement and changes in population dynamics.

Large wildfires occurring in recent years have led to international and global impacts, which could pose unforeseen long-term problems. Transboundary pollution such as smoke and pollutants from wildfires can cross national borders, affecting neighboring countries and even continents. This can lead to international tensions and the need for cross-border cooperation on air quality management [14,29]. Additionally, large-scale wildfires can influence global climate patterns, potentially affecting weather systems and contributing to phenomena such as El Niño and La Niña [30,31,32,33,34,35,36,37,38]. For example, the event of the 2019–2020 Australian bushfires is known as the “Black Summer,” with smoke from the fires traveling across the Pacific Ocean and even affecting the air quality as far away as South America and contributing to carbon emissions that impacted global climate patterns. The smoke from the 2017 British Columbia wildfires spread across Canada and into the United States, affecting the air quality in cities like Seattle, Portland, and even as far east as Denver.

Addressing the impacts of intercontinental wildfires and the human health risks associated with exposure requires coordinated international efforts. Chemical exposure, a part of the total human exposome, is a challenge to assess because of the abundance of chemicals and their levels of exposure for each scenario. Risk assessment methods provide a paradigm for assessing the risk to humans from exposure to these chemicals. The Environmental Protection Agency (EPA) provides a paradigm for human health risk assessment to assess exposure to chemicals in the environment. It uses risk assessment to describe the nature and extent of risks to human health for various populations, including workers and sensitive subpopulations. The risk assessment process is iterative and helps to identify factors that play the most significant role in the calculated risk. The following is a brief summation of the EPA paradigm for inhalation risk assessment.

The EPA’s Superfund Program updated its previous approach for determining the risk from inhaled chemicals to be consistent with the inhalation dosimetry methodology described in the Methods for Derivation of Inhalation Reference Concentrations and Application of Inhalation Dosimetry [39]. The document provides risk assessors with guidance that more consistently addresses the inhalation dosimetry methodology. Included in the document are recommended processes consisting of a series of steps as well as recommended equations. The guidance is intended to provide a recommended methodology for consistently addressing the inhalation pathway in risk assessments for not only Superfund sites but also across other sites with inhalation exposure. This guidance is readily applied to any inhalation scenario, such as the scenarios highlighted in this study.

This study focused on evaluating the risks associated with an intra-continental transport event from wildfires occurring in July and August 2021, a major wildfire crisis since 2020 characterized by intense and widespread fires that severely impacted the air quality across North America. The series of intense wildfires was driven by extreme drought conditions and high temperatures. Wildfire smoke from Canada and the western U.S. was carried by the jet stream and cross-continental winds and spread across the country, worsening the air quality and visibility from the west to the east coasts. The enhanced smoke exposure and health risks across the U.S. need an accurate quantification and assessment. We specifically focused on PM with a diameter of 2.5 μm and smaller (PM_2.5_), which is a key indicator of the air quality and has been the primary pollutant of health concern from wildfires.

While much research has concentrated on local and regional impacts, this study fills a critical gap by emphasizing the intra-continental transport of wildfire smoke across North America, specifically examining how smoke from the western U.S. and Canada travels, affecting vast regions far beyond the fire’s immediate vicinity, including the eastern U.S. and Canada. This broad focus offers a more comprehensive understanding of wildfire smoke’s geographic reach and provides a multi-country perspective that highlights the cross-border implications policymakers, public health officials, and emergency responders must consider. Additionally, while many studies have explored wildfire smoke’s health effects or atmospheric dynamics independently, this study bridged that gap by integrating advanced atmospheric modeling with health risk assessments, linking GEOS-Chem’s high-resolution simulations with health exposure metrics to offer a holistic view of how long-range smoke transport contributes to public health risks, particularly in regions not traditionally considered at risk. Going beyond previous studies that estimated the health impacts for specific areas, this research assessed the health risks for large populations, especially in densely populated urban centers. The innovation of this study lies in its unique interdisciplinary approach, combining atmospheric modeling and epidemiological data to track the long-range transport of smoke and quantify its associated health risks, providing a comprehensive analysis of exposure across large regions. By taking advantage of recent large wildfires with significant cross-border effects, this study offers an unprecedented multi-country perspective on the health impacts of wildfire smoke on a vast, diverse population across North America [40,41,42,43,44,45].

## 2. Methods

### 2.1. GEOS-Chem Modeling of Wildfire-Induced Aerosol Enhancements

We used GEOS-Chem, a global 3D chemical transport model (version 13.4.1; https://wiki.seas.harvard.edu/geos-chem/index.php/GEOS-Chem_13.4.1, last accessed on 17 April 2024) in this study to simulate the long-distance transport of wildfire smoke that occurred in July and August of 2021 and the changes it induced in the concentrations of PM_2.5_ over North America. The GEOS-Chem model was chosen for this study due to its comprehensive capability to simulate atmospheric compositions from local to global scales. We used GEOS-Chem in the off-line mode (i.e., GEOS-Chem Classic), driven by assimilated meteorological data from the Goddard Earth Observation System (GEOS) of the NASA Global Modeling and Assimilation Office (GMAO) on a rectilinear latitude–longitude grid, to compute the horizontal and vertical transport. GEOS-Chem modeling accounts for processes such as emissions, transport, deposition, radiation, chemistry, and aerosol dynamics, integrating detailed meteorological data and pollutant emission inventories. Given the large domain targeted in this study, the high-resolution output from GEOS-Chem allowed for the accurate modeling of long-range smoke transport and its effects on the air quality, which are crucial for assessing exposure and health risks.

Particle constituents were also simulated, but their risk analysis was beyond the scope of this study. Two sets of GEOS-Chem simulations were performed, with one simulation driven by the Global Fire Emissions Database version 4.0 (GFED4) [46] and a parallel simulation without fire emissions serving as a reference; thus, the differences between the two simulations with and without fire emissions indicated the fire-induced enhancements in air pollutants. The GFED4 provides global monthly and daily burned area data at a 0.25° spatial resolution from 2000 through to the present, with the data produced by combining 500 m MODIS burned area maps with active fire data from the Tropical Rainfall Measuring Mission (TRMM) Visible and Infrared Scanner (VIRS) and the Along Track Scanning Radiometer (ATSR) satellite sensors [46]. In addition, other emissions inventories such as global anthropogenic emissions from the Community Emissions Data System version 2 (CEDSv2) as well as biogenic emissions from the Model of Emissions of Gases and Aerosols from Nature version 2.1 (MEGAN2.1) were used, allowing the all-emission simulation to reproduce realistic atmospheric conditions that were influenced by a range of sources [47,48]. For each scenario, we first performed a global simulation at a 4° latitude × 5° longitude spatial resolution. The global results were then used as boundary conditions in the downscaled nested simulation that covered the North American domain, with a latitudinal range of [9.75, 60] and longitudinal range of [−130, −60] at a 0.25° × 0.3125° resolution. The NASA Global Modeling and Assimilation Office (GMAO)’s GEOS-FP (“forward-processing”) meteorological data product was used to drive the GEOS-Chem meteorology. Global simulations included an extra spin-up year and nested simulations used an extra spin-up month to generate reasonable initial physics and dynamics states for each scenario.

Wildfires release a variety of trace gasses and PM into the atmosphere, many of which have significant environmental and health impacts. GEOS-Chem incorporates the impacts of processes such as advection, diffusion, and turbulent mixing on pollutant transport across different model grids. We focused on the health risk of PM in this study. For computational efficiency, we used the aerosol-only version of GEOS-Chem, with monthly mean oxidants archived from a full-chemistry simulation, as described by Park et al. (2004) and used in our previous wildfire study [49]. Building on previous studies, 35% of biomass burning emissions by mass are distributed across the 10 sigma layers above the boundary layer. This is especially relevant for large wildfires, such as those examined in this study, which could result in a significant injection height [49,50,51]. The modeled results from this all-emission simulation were compared with ground-based observational data from the Interagency Monitoring of PROtected Visual Environments (IMPROVE) and AirNow in the U.S. and the National Air Pollution Surveillance (NAPS) program in Canada for validation and evaluation.

### 2.2. Human Health Risk Assessment

The historic guidance given in the EPA’s Risk Assessment Guidance for Superfund (RAGS), Part A, outlines a previously recommended approach for conducting site-specific baseline risk assessments for inhaled contaminants. According to the original RAGS approach, the inhalation exposure estimate was typically derived in terms of a chronic, daily “air intake” (mg/kg-day) using the following general approach. The intake of the chemical was estimated as a function of the concentration of the chemical in air (CA), the inhalation rate (IR), the body weight (BW), and the exposure scenario. Age-specific values for the BW and IR were used when evaluating specific exposure scenarios (e.g., asthmatics) [52].

Table 1 presents the RAGS, Part A, equation for calculating the intake for the inhalation exposure. Inhalation toxicity values were “converted” into similar units for the risk quantification step. The cancer risk was estimated by multiplying the chronic daily intake of the chemical from the air by the “inhalation cancer slope factor” (CSFi); the hazard quotient (HQ) for non-cancer effects was estimated by dividing the intake of the chemical by an “inhalation reference dose” (RfDi) [52]. The approach outlined in RAGS, Part A, was developed before the EPA issued the inhalation dosimetry methodology, which describes the Agency’s refined recommended approach for interpreting inhalation toxicity studies in laboratory animals or studies of the occupational exposure of humans to airborne chemicals.

Updated inhalation dosimetry guidance from the EPA recommends that when estimating the risk via inhalation, risk assessors should use the concentration of the chemical in the air as the exposure metric (e.g., mg/m^3^), rather than the inhalation intake of a contaminant in the air based on the IR and BW (e.g., mg/kg-day).

The intake equation described above (RAGS, Part A, Exhibit 6–16) is not consistent with the inhalation dosimetry methodology because the amount of the chemical that reaches the target site cannot be described simply using ingestion rates and the body weight. Rather, the complex interaction of the inhaled contaminant with the respiratory tract is affected by many factors, such as the species-specific relationships of the exposure concentrations (ECs) to the deposited/delivered doses in various parts of the airway and lung and the physiochemical characteristics of the inhaled contaminant [53,54,55,56]. The inhalation dosimetry methodology also considers the target site where the toxic effect occurs (e.g., the respiratory tract or a location in the body remote from the portal of entry) when applying dosimetric adjustments to experimental concentrations [39,57,58,59]. Therefore, the RAGS, Part A, equation, illustrated in Table 1, is not recommended for estimating the exposure to inhaled contaminants. In this study, we used the inhalation dosimetry methodology recommended by the USEPA to evaluate the risk to adults, children, and wildfire firefighters.

In general, a human health risk assessment is a four-step process including hazard identification, toxicity assessment, exposure assessment, and risk characterization. The purpose of hazard identification is to determine the chemicals of concern for the affected population. Toxicity assessment is accomplished through the evaluation of dose–response relationships, which is mainly performed through animal studies and their results are generalized to humans. The evaluation of the exposure is accomplished using parameters such as the amount and duration of exposure that are specific to the scenario of the exposure. After analyzing data on the exposure and toxicity, the level of risk posed by the chemical is determined and compared to acceptable levels set forth by the EPA, and appropriate corrective measures are recommended in the risk characterization.

After determining the concentration of chemicals in the air, and based on the exposure patterns identified, the concentration of the exposure is then determined as follows.

A: Chronic and subchronic exposure:

(1) $$EC=\frac{\left(CA\times ET\times EF\times ED\right)}{AT}$$

B: Acute exposure:

(2) EC=CA

EC: exposure concentration (µg/m^3^).
CA: contaminant concentration in the air (µg/m^3^).
ET: exposure time (hours/day).
EF: the exposure frequency, which indicates the number of exposure days per year (days/year).
ED: exposure duration in years.
AT: average time (hours) (the period over which the exposure is averaged in terms of days; ED (years) × 365 (days/year) × 24 (hours/day)).

If the exposed population does not have a uniform activity pattern, like wildfire firefighters, the following formula is used to calculate the exposure concentration in that microenvironment for a specific time period:

(3) $${EC}_{j}=\sum_{i=1}^{n}\frac{({CA}_{i}\times {ET}_{i}\times {EF}_{i})\times {ED}_{j}}{{AT}_{j}}$$

where

EC_j_: average exposure concentration for a certain exposure period, j (µg/m^3^).
CA_i_: contaminant concentration in the air in the microenvironment i (µg/m^3^).
ET_i_: exposure time spent in microenvironment i (hours/days).
EF_i_: exposure frequency for microenvironment i (days/year).
ED_j_: exposure duration for exposure period j (years).
AT_j_: average time (hours) (the period over which the exposure is averaged in terms of days; ED (years) × 365 (days/year) × 24 (hours/day)).

When the population is exposed to varying concentrations of chemicals over different periods, the average concentration over the long term is calculated using the following equation:

(4) $${EC}_{LT}=\;\sum \frac{\left({EC}_{j}\times {ED}_{i}\right)}{AT}$$

where

EC_LT_: long-term average exposure concentration (µg/m^3^).
EC_j_: average exposure concentration of the contaminant in the air for exposure period j (µg/m^3^).
ED_j_: exposure duration in period j (years).
  AT: averaging time (years).

The evaluation of the toxicity of a chemical substance is conducted using toxicity reference benchmarks, namely the inhalation unit risk for carcinogens and the hazard quotient for non-carcinogenic effects. After finding the inhalation unit risk (IUR), the amount of carcinogenic risk of a substance is determined using the following formula:

(5) Risk=EC×IUR

where

EC: exposure concentration (µg/m^3^).
IUR: carcinogenic unit risk (µg/m^3^).

For non-carcinogenic risk calculations, a hazard quotient (HQ) is calculated. The hazard quotient represents the concentration below which there would be a negligible risk of a non-carcinogenic effect.

(6) $$HQ=\;\frac{EC}{Toxicity\;Value\;\left(i.e.,\;RfC\right)\times 1000\;\mu g/mg}$$

where

HQ (unitless) = hazard quotient.
EC (μg/m^3^) = exposure concentration.
Toxicity Value (mg/m^3^) = inhalation toxicity value (e.g., RfC) that is appropriate for the exposure scenario (acute, subchronic, or chronic).

After calculating the risk for both carcinogens and non-carcinogens, the amount is compared with the acceptable risk limits. For carcinogens, an acceptable risk level is defined as less than 1 in 1 million persons developing cancer from exposure. For non-carcinogenic risks, a sum of hazard quotients below 1 is considered acceptable.

## 3. Results

### 3.1. Model–Observation Comparison and Limitations of IMPROVE Observations

**Figure 1:**
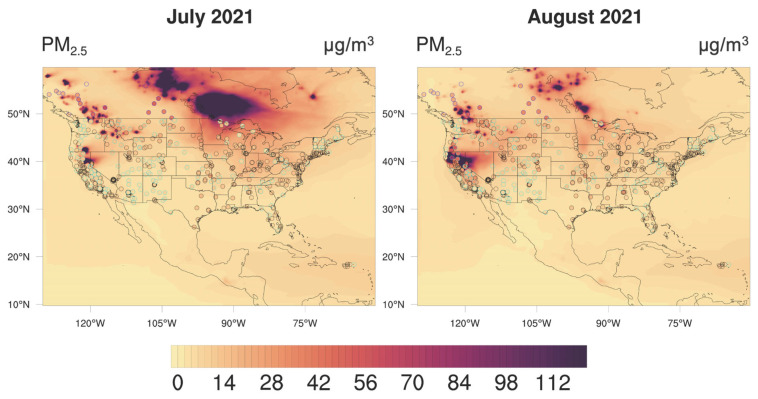
Model–observation comparison of the spatial distributions of surface PM_2.5_ concentrations averaged over July (**left panel**) and August (**right panel**) 2021. The monthly averaged GEOS-Chem results, driven by all emissions, are shown as the contour background, while observations from three monitoring networks are represented by colored dots. IMPROVE sites are marked with cyan-circled dots, AirNow sites with black-circled dots, and NAPS sites with blue-circled dots.

The GEOS-Chem-modeled results over the U.S. and southern Canada from the all-emission simulation were evaluated against ground-based observations from the IMPROVE, AirNow, and the NAPS. The PM_2.5_ concentrations measured at these observational sites were compared with the modeled concentrations in the corresponding grid cells covering the same geographical locations (Figure 1). The IMPROVE data are obtained every three days, and the sampling sites cover most of the states in the U.S. The spatial distributions of PM_2.5_ concentrations in July and August 2021, as observed by the IMPROVE, indicate enhancements in the western, northern, and central U.S. regions, with the spatial pattern of the enhancements captured by GEOS-Chem modeling, despite the model showing a significant overestimation. Daily average AirNow and NAPS data from sites with both PM_2.5_ and PM_10_ measurements were used, as comparing these two air pollution indices helped ensure the data quality. For example, we excluded sites where the measured PM_2.5_ concentrations exceeded the PM_10_ levels. AirNow provides denser coverage across the U.S. than the IMPROVE and better captures the spatial distribution of PM_2.5_ concentrations compared to both the IMPROVE and NAPS.

**Figure 2:**
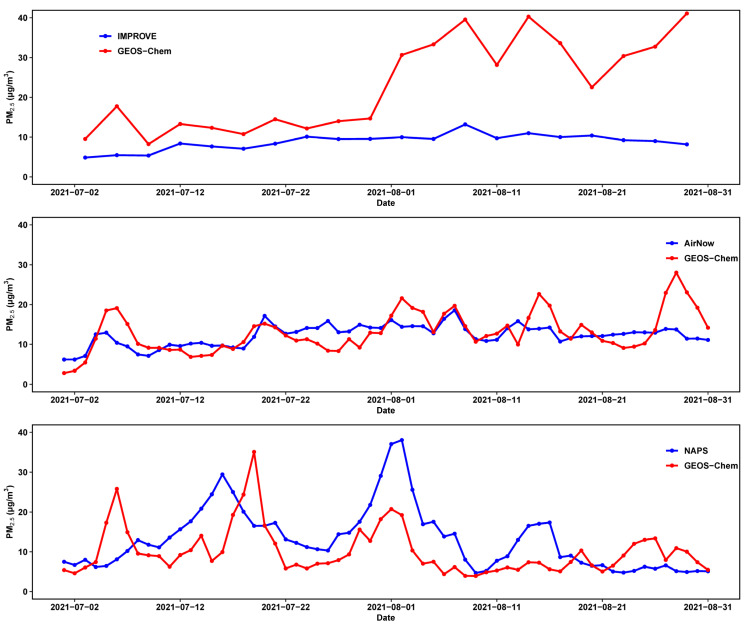
Timeseries comparison of PM_2.5_ concentrations between IMPROVE observations and GEOS-Chem outputs, averaged across all IMPROVE sites and their corresponding GEOS-Chem grid cells (**upper panel**); between AirNow and GEOS-Chem data, averaged across all AirNow sites and their corresponding GEOS-Chem grid cells (**middle panel**); and between NAPS and GEOS-Chem data, averaged across all NAPS sites and their corresponding GEOS-Chem grid cells (**lower panel**).

The timeseries comparison of PM_2.5_ concentrations between the IMPROVE and GEOS-Chem showed noticeable observation–model discrepancies, especially over August, where GEOS-Chem simulated significant increases in PM_2.5_, much higher than the IMPROVE observed values (Figure 2). While observations are typically used as benchmarks to validate model results, it is important to note that IMPROVE sampling and measurements could be constrained by their upper limit of detection. This limitation may become particularly relevant during periods of large wildfires when ambient concentration levels experience sharp increases, potentially causing the filters to reach their maximum sampling capacity. For example, at the IMPROVE Bliss State Park (BLIS) sampling site in South Lake Tahoe, CA, on 29 August 2021, the GEOS-Chem-simulated PM_2.5_ concentrations reached up to 491.15 µg/m^3^, whereas the IMPROVE measurements resulted in an error. IMPROVE data also showed stable values under conditions of high pollution. At the BLIS sampling site, another example is 8 August 2021, when the daily average GEOS-Chem PM_2.5_ concentration reached 327.93 µg/m^3^, while the IMPROVE recorded 44.03 µg/m^3^ on the same day. A notable maximum GEOS-Chem PM_2.5_ concentration of 2414.65 µg/m^3^ was recorded on 29 August 2021, whereas the corresponding IMPROVE PM_2.5_ concentration was 47.99 µg/m^3^. This analysis underscores the need for caution when interpreting IMPROVE observational data for extreme pollution events. However, this issue is not well documented to our knowledge.

Despite discrepancies between the GEOS-Chem-simulated PM_2.5_ concentrations and IMPROVE observations, measurements from AirNow sites better reproduced the variations in PM_2.5_ over July and August (Figure 2). In southern Canada, GEOS-Chem slightly underestimated PM_2.5_ but captured its variability well, compared with NAPS observations. It is important to note that while preliminary data quality assessments have been conducted, AirNow data have not undergone full validation, which may introduce biases in the model–observation comparison. Additionally, NAPS data products are subject to ongoing updates, which could also contribute to model–observation discrepancies. However, we noted that the modeled surface smoke from GEOS-Chem closely aligned with the magnitude and distribution of and variation in near-surface smoke levels captured by the High-Resolution Rapid Refresh Smoke (HRRR-Smoke) model across the U.S. and southern Canada over the two months (https://rapidrefresh.noaa.gov/hrrr/HRRRsmokeold/, last accessed on 13 June 2024). By comparing the GEOS-Chem model results with multiple observational datasets and the HRRR model, we could more reliably use the model results to assess the health risks associated with the worsened air quality induced by wildfires. Given that GEOS-Chem tended to underestimate PM concentrations compared to the AirNow and NAPS datasets, our health risk assessments may have been biased low.

### 3.2. Evolution of Wildfire-Derived PM_2.5_

**Figure 3:**
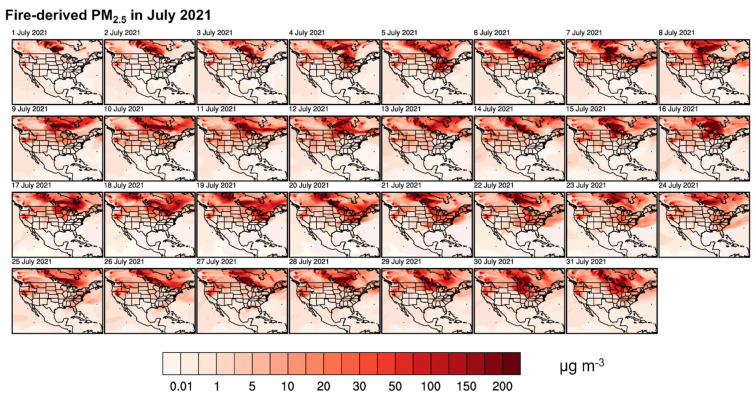

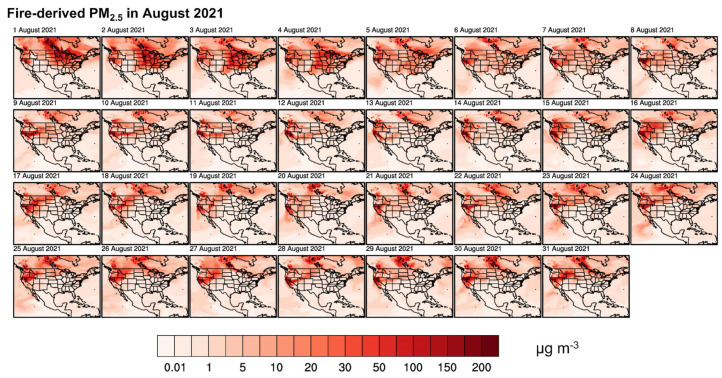
Evolution of daily average wildfire-derived surface PM_2.5_ concentrations in July (**upper panel**) and August (**lower panel**) of 2021.

We calculated fire-induced enhancements in the PM_2.5_ based on the differences between the two GEOS-Chem simulations with and without fire emissions. The evolution of the spatial distributions of the daily average wildfire-derived PM_2.5_ concentrations in July and August are shown in Figure 3. During July and August 2021, a series of intense wildfires across the western U.S., particularly in California, Oregon, and Washington, combined with significant wildfire activity in Canada, particularly in British Columbia and Alberta, led to the widespread intra-continental transport of smoke. These fires were fueled by extreme drought conditions, high temperatures, and strong winds, creating a perfect storm for large-scale smoke dispersion across North America.

In the western U.S. and Canada, the areas closest to the wildfires, including the Pacific northwest in the U.S. and western Canada, experienced the highest concentrations of PM_2.5_. Cities like Vancouver, Seattle, and Portland recorded extremely poor air quality, with PM_2.5_ reaching hazardous levels for extended periods. The proximity of the fires led to dense smoke and significantly reduced air quality across the region. As the smoke plumes from both the U.S. and Canadian wildfires traveled eastward, states in the midwest, including Colorado, Nebraska, and Illinois, as well as provinces like Manitoba and Ontario in Canada, observed elevated PM_2.5_ levels. The convergence of smoke from multiple sources led to widespread air quality degradation in these areas.

In early July, wildfires in California, Oregon, British Columbia, and Alberta intensified, producing large quantities of smoke. The smoke initially remained localized but began spreading eastward by mid-July. The air quality in the western U.S. and Canada rapidly deteriorated, with PM_2.5_ reaching unhealthy levels. During mid-July, the smoke continued to move eastward, with elevated PM_2.5_ levels detected across the central US and Canada. During this time, the influence of the smoke extended into the Great Lakes region and the central provinces of Canada, leading to hazy skies and moderate air quality concerns. By late July, the smoke had reached the eastern states, affecting the air quality from the midwest to the Atlantic coast and from Ontario to the maritime provinces. The PM_2.5_ concentrations in New York, Pennsylvania, and the mid-Atlantic region, as well as the eastern provinces of Canada, such as Quebec and Nova Scotia, were even comparable to the those of the western regions, significantly degrading the air quality.

The elevated concentrations of PM_2.5_ over eastern North America lasted until early August as the wildfire activity persisted in both the U.S. and Canada, and the smoke plumes continued to affect large areas of North America. The central and eastern regions experienced recurring episodes of poor air quality as the smoke was transported repeatedly across the continent. As the wildfire activity began to decline in mid-to-late August, the air quality gradually improved. However, residual smoke lingered in the atmosphere, and sporadic increases in PM_2.5_ levels were observed, especially during periods of strong winds that re-entrained smoke particles into the air.

The persistently elevated PM_2.5_ levels in both July and August prompted health advisories from the west to the eastern seaboard of both countries. Overall, the smoke dispersion followed a pattern of episodic spikes in the PM_2.5_ levels, corresponding to the peaks of wildfire activity in both the U.S. and Canada. In July, the smoke transport was more concentrated, while in August, the combined effects of continued fires in both countries led to widespread and persistent smoke, affecting large portions of North America simultaneously.

### 3.3. Human Health Risk Associated with Fire-Derived PM_2.5_

Using the EPA Inhalation Risk Paradigm (RAGS Part F) to determine the empirical health risk to evaluate the enhanced health risks across the nation for specific populations, we evaluated the risk associated with PM_2.5_ exposure for the following groups:

◦ Adults.
◦ Children 0–16 years of age.
◦ Children 16–18 years of age.
◦ Firefighters.

The exposure factors used for each population group to calculate the exposure concentration (Equation (1)) are presented in Table 2.

After characterizing the exposure scenarios and estimating the exposure concentrations as described above for each receptor at a site, the next step was selecting appropriate inhalation toxicity values for each inhaled contaminant. Typically, for estimating cancer risks, this involves identifying and evaluating the available published cancer potency estimates, the inhalation unit risk factors (IURs). For estimating non-cancer risks or hazard quotients, this typically involves identifying and evaluating reference values (RfCs) that match the characterization of the exposure scenario (i.e., acute, subchronic, or chronic reference values).

These values are typically published in the Integrated Risk Information System (IRIS), which is updated by the EPA. However, for PM, there are not any published IURs or RfCs. This means that risk assessors must go to the scientific literature to evaluate dosimetry studies and estimate preliminary toxicity values to estimate risk. With a lack of consensus regarding the Reference Concentration (RfC) of PM_2.5_, we used the 5 μg/m^3^ RfC of diesel particles (DPM) that has been established in the literature as a toxicity surrogate for PM_2.5_ [60,61,62]. Similarly, the inhalation unit risk values were obtained from the literature for PM_2.5_. The IUR for PM_2.5_ is 0.008 [9,15,63,64,65].

Additionally, when calculating the carcinogenic risk, age adjustments should be considered for those carcinogens that have been determined to cause cancer by a mutagenic mode of action, as it is possible that exposure to those carcinogens in early life may result in higher lifetime cancer risks than a comparable adult exposure duration [66]. Some studies have shown that PM can mutate cells, causing lung cancer [67,68]. In risk assessments of the exposure to chemicals for which a mutagenic mode of action for carcinogenicity is suspected or has been determined, one of the following generally guides the calculations:

(1) If chemical-specific data on susceptibility from early-life exposure are available for the derivation of CSFs, those slope factors are used for risk characterization, and Age-Dependent Adjustment Factors (ADAFs) are not applied.
(2) If chemical-specific data on susceptibility from early-life exposure are not available, ADAFs are applied in calculating or estimating the risks associated with early-life exposure [69]. If the latter case applies, as in this study, the Supplemental Guidance for Assessing Susceptibility from Early-Life Exposure to Carcinogens [70] recommends the following default ADAFs be applied in risk assessments:

- A 10-fold adjustment for exposure during the first 2 years of life;
- A 3-fold adjustment for exposure from 2 to <16 years of age;
- No adjustment for exposure after turning 16 years of age.

In such cases, Equation (5) is altered to include the ADAFs in the following way:

(7) Risk=IUR×EC×ADAF

**Figure 4:**
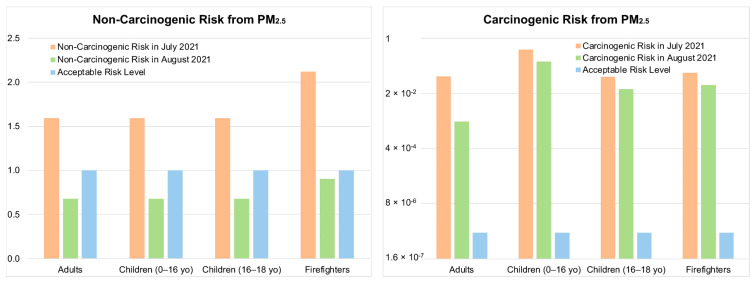
Estimated non-carcinogenic and carcinogenic risk associated with exposure to average fire-derived PM_2.5_ concentration across the North American domain for specific populations during July and August of 2021.

For this study, the ADAF was conservatively set at 7. The average concentration of fire-derived PM_2.5_ was 16.62 µg/m^3^ in July and 7.07 µg/m^3^ in August. The risks associated with fire-sourced PM_2.5_ are presented in Figure 4.

In July, the non-carcinogenic risk was calculated to be above the acceptable limit of 1 for each receptor evaluated. Similarly, the carcinogenic risk was orders of magnitude above the acceptable limit of one in one million (<1 × 10^−6^) in July. The carcinogenic risks were elevated for all groups and, in particular, young children and wildfire firefighters were at very high risk in the month of the active fires. In August, the risk profiles for the non-carcinogenic risk were trending below the acceptable threshold. However, firefighters were only slightly under the acceptable risk levels. All receptors were close to the unacceptable risk threshold, indicating that susceptible populations (i.e., asthmatics) could still be facing non-carcinogenic health effects. The carcinogenic risks in August remained significantly higher than the acceptable limit for all groups, and once again these data indicate that young children and wildfire firefighters were two particularly sensitive populations impacted, even in August, when the fire activities were largely reduced compared to July.

When comparing the present study to previous studies in the scientific literature, we must look at comparable levels of exposure [71]. The comparative studies are sparse at best; however, comparable levels of risk are associated with heavy smokers [72,73]. For example, air quality index values (AQI) on a normal day in LA are between 50 and 60. However, on a smoky day these values go up to 100–200, and during the LA wildfires these values reached 400 and were above 500 many times. The risks associated with the exposure to PM at an AQI of 100–200 are comparable to smoking a half-pack of cigarettes a day (approximately 10–12 cigarettes), whereas an AQI of 400 is like smoking a pack or more (23+ cigarettes) a day [74,75]. In a study evaluating the health risks of chronic smoke exposure for wildland firefighters, 15 substances in smoke were evaluated. In that study, only benzene and formaldehyde posed a cancer risk greater than 1 per million, while only acrolein and respirable particulate matter exposure resulted in hazard indices greater than 1.0. The estimated upper bound cancer risks ranged from 1.4 to 220 excess cancers per million, and the non-cancer hazard indices ranged from 9 to 360, depending on the exposure group [75]. Our study specifically evaluated the contribution of the risk from fine particulate matter, not considering other toxic components in wildfire smoke, and the risk associated with the particulate matter alone was as high as some of those seen in the aforementioned study.

## 4. Conclusions

This study simulated the evolution of wildfire-induced aerosols and evaluated the impact of wildfires on human health from fire-derived PM_2.5_ exposure during intra-continental wildfire events that occurred in July and August 2021. The GEOS-Chem model was used, and the model results were compared with observational datasets from the IMPROVE, AirNow, and NAPS networks. Although observations are typically used as benchmarks to validate model results, we found that IMPROVE sampling and measurements could be constrained by their upper limit of detection, especially during periods of large wildfires when ambient concentration levels experience sharp increases. However, the upper bounds of IMPROVE measurements are not documented to our knowledge. Despite discrepancies between the GEOS-Chem-simulated PM_2.5_ concentrations and IMPROVE observations, the GEOS-Chem-modeled surface smoke closely aligned with near-surface smoke levels simulated by the HRRR-Smoke model across the U.S. and southern Canada. In addition, the GEOS-Chem-simulated PM_2.5_ concentrations closely matched those observed by AirNow in the U.S. However, over southern Canada, the PM_2.5_ concentrations were underestimated by GEOS-Chem compared to NAPS data, though the general variability was well reproduced.

Through GEOS-Chem modeling, we could effectively identify elevated PM levels induced by wildfires. The persistently elevated levels of PM_2.5_ throughout July and August necessitated health advisories across both the U.S. and Canada, from the west coast to the eastern seaboard. The pattern of smoke dispersion revealed episodic spikes in particulate matter, aligning closely with periods of intensified wildfire activity in both countries. Specifically, July exhibited more concentrated smoke transport, whereas August saw a broader and more persistent impact due to ongoing fires. This widespread and prolonged smoke exposure underscores the need for ongoing vigilance and public health measures to mitigate the health risks associated with elevated particulate matter levels across North America.

Due to the significant impacts from intra-continental wildfire smoke transport on human health and considering the anticipated variations in wildfire compositions due to climate change, there is a pressing need to better understand and address these health risks. Our study identified substantial carcinogenic risks associated with exposure to PM_2.5_ over a two-month period for various populations, including adults, children, and firefighters. These risks were five orders of magnitude higher in some cases (e.g., for children 0–16 yo) than the acceptable cancer risk threshold of one in one million persons. The findings highlight that young children and wildfire firefighters are particularly sensitive groups, underscoring the necessity for targeted interventions and preventive measures. Additionally, the limitations of IMPROVE observations are further highlighted in the risk assessment results. If IMPROVE observations are underestimating the actual concentrations of PM_2.5_ in the atmosphere, the associated health risk derived based on this observational data could be underestimated. As previously noted, the observed variations in the maximum concentrations across all IMPROVE sites over the two-month period suggest that high-smoke events may not be fully captured. Consequently, risk estimates derived from these data might significantly underrepresent the adverse health effects experienced by exposed individuals.

## 5. Future Work

Understanding the dynamics of chemical adsorption onto particulate matter during wildfires is crucial for developing effective strategies to protect both human health and the environment. This further illustrates the need to study wildfire compositions and the predicted changes that atmospheric movement and climate change may bring, which could further elevate human health risks.

Future studies must prioritize incorporating mixture toxicology to assess the health impacts of chemicals adsorbed onto particulate matter produced during wildfires, as well as evaluating the risks posed to underrepresented communities residing in areas affected by wildfires. Sensitive subpopulations stand to endure greater impacts than the general populations evaluated in this study. Characterizing contaminants more robustly using advanced modeling techniques, such as those used in this study, will enable better predictions of changes in wildfire particulate and chemical compositions over time, and developing improved methods to incorporate risk assessments for all receptors, including sensitive subpopulations, will significantly enhance our ability to protect both human health and the environment.

## Figures and Tables

**Table 1:** Risk Assessment Guidance for Superfund (RAGS), Part A, equation describing the estimation of the inhalation exposure, Chapter 6, Exhibits 6–16, Pages 6–44.

Intake (mg/kg-day) =	CA × IR × ET × EF × ED
	BW × AT
CA =	chemical concentration in air (mg/m^3^)
IR =	inhalation rate (m^3^/hr)
ET =	exposure time (hours/day)
EF =	exposure frequency (days/year)
ED =	exposure duration (years)
BW =	body weight (kg)
AT =	averaging time (days)

**Table 2:** Exposure factors used to calculate exposure concentrations for specific populations.

	Adults	Children (0–16 Years Old)	Children (16–18 Years Old)	Firefighters
Exposure duration (ED)—years	80	16	3	30
Exposure frequency (EF)—days/year	350	350	350	350
Exposure time (ET)—hours/day	12	12	12	16
Averaging time (AT)—hours	700,800	140,160	26,280	262,800

Averaging time = ED in years × 365 days/year × 24 h/day.

## Data Availability

The raw data supporting the conclusions of this article will be made available by the authors on request.
